# Supplementary material for: A Normative Model Representing Autistic Individuals Amidst Autism Spectrum Phenotypic Heterogeneity
Source: Brain Sci. 2024 Dec 14;14(12):1254. doi: 10.3390/brainsci14121254 (PMC11674926; doi:10.3390/brainsci14121254)
Supplement: Supplementary file 1 [file brainsci-14-01254-s001.zip › brainsci-3355736-supplementary.pdf]

Supplementary Material

Supplementary Methods

IQ Quantification Details for SSC

For Brazilian patients' cognitive assessment the Total Intelligence Quotient (IQ) was selected (Laros et al. 2013 "Brazilian validation of the nonverbal intelligence test SON-R 2½-7[a]", Karino et al. 2011 "Evidences of convergent validity of SON-R 2½-7[a] with WPPSI-III and WISC-III") since it provides standardized and age-corrected estimates of overall cognitive ability validated in Portuguese language.

Normative Modeling

A 3-dimensional Gaussian distribution (also known as Multivariate Normal distribution) was fitted to the PCA-derived vector space as usual. The actual data used to fit the model are not raw PCA results but rather a Z-score-like normalized vector space truncated (projected) at 3 dimensions. Estimates for PC1, PC2 and PC3 dimension's means and standard deviations were used to normalize the observation coordinates in terms of "standard deviations from the mean". This is the final 3D presentation in which the normative modeling is performed. Observed covariances among all three dimensions were approximately zero and forced as such. Therefore, the fitted model can be reduced to independent univariate N(0,1) normal distributions in each of the 3 principal component axes. The multivariate probability density function is spherically symmetrical.

The SSC (normative population) estimated parameters, 3 pairs of means and standard deviations, are used to transform any new observation from the original PCA-rotated space to the standardized space.

Due to its spherical symmetry, the 3-dimensional probability density function can be easily rotated in any direction, in particular, the defined "support needs'" axis which goes from the all negative octant to the all positive octant. Therefore we can calculate the theoretical quantile of a given point in space simply using a straightforward N(0,1) integral:

$$\mathrm{M} = \int_{-\infty}^{q} \int_{-\infty}^{\infty} \int_{-\infty}^{\infty} (2\pi)^{\frac{-3}{2}}\, e^{\frac{-1}{2}\,(r_1^2 + r_2^2 + r_3^2)}\, \mathrm{d}r_1\, \mathrm{d}r_2\, \mathrm{d}r_3 \tag{S1}$$

$$\mathrm{M} = \int_{-\infty}^{q} \frac{1}{\sqrt{2\pi}}\, e^{\frac{-1}{2}r^2}\, dr \tag{S2}$$

where r_1, r_2 and r_3 are the coordinates of a patient in a rotated 3-dimensional coordinate system that aligns $r_3 = r$ along the support needs' axis, which in turn follows the main diagonal of direction (1,1,1) in the plot. Using this symmetry argument, the value q is just the distance between the plane $Ax_1 + Bx_2 + Cx_3 + D = 0$ orthogonal to the axis which contains the 3-dimensional point representing the patient:

$$q = \frac{D}{\sqrt{A^2 + B^2 + C^2}} \tag{S3}$$

where x_1, x_2 and x_3 are the new patient coordinates in the plot (thus Z-score normalized PCA space 3-dimensional projection).

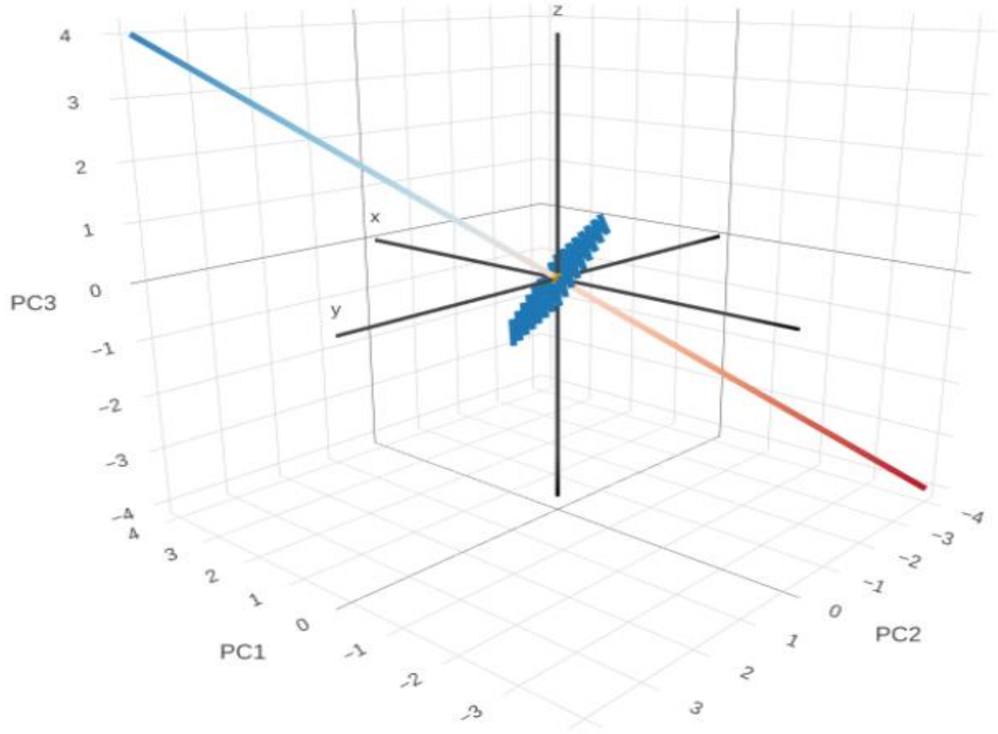

Figure S1. Ax_1 + Bx_2 + Cx_3 + D = 0 plane (blue) normal to the support needs' axis (diagonal red-to-blue line) is shown in the plot. In this specific example, the origin (0,0,0) lies in the plane and, therefore: M = 0.50 = 50% leads to MGFS = 5.0 and q = 0, since it splits the spherically symmetric multivariate normal probability density function in exactly half.

The Equation (2) returns the cumulative probability of all observations up to the new point for which we want to determine a deviation index concerning the reference sample (MGFS):

$$\text{MGFS} = 10\ \text{M} \tag{S4}$$

Supplementary Tables

Table S1. Means and standard deviations from the scores obtained by patients with ASD from the SSC sample in the tests, scales, and questionnaires used as input for the PCA.

**ADI-R**	
Socialization	20.32 (5.70)
Communication	15.95 (4.31)
Restricted and repetitive behavior	6.52 (2.49)
**Full-scale IQ**	
IQ	81.23 (27.9)
**VABS**	
Communication	77.12 (14.49)
Socialization	70.98 (12.55)
Daily Living Skills	76.45 (13.83)
**CBCL Problems Índex** *	
Internalizing Problems	60.31 (9.55)
Externalizing Problems	56.57 (10.6)
* Data are T-scores	

Table S2. Means and standard deviations of the scores obtained by the case presentation patients in the tests, scales and questionnaires.

ADI-R	
Socialization	22.81 (4.09)
Communication	12.52 (3.7)
Restricted repetitive behavior	5.85 (2.46)
**SON-R**	
IQ	58.74 (8.96)
**VABS**	
Communication	46.7 (6.92)
Socialization	55.33 (8.09)
Daily Living Skills	50.85 (13.16)
**CBCL Problems Índex ***	
Internalizing Problems	67.85 (8.75)
Externalizing Problems	64.59 (8.89)
* Data are T-scores	

Table S3. Variance explained by each principal component when using the sub-items of VABS, ADI-R, total IQ and internalizing and externalizing CBCL evaluated in 2744 SSC patients as input measures.

Components	1	2	3	4	5	6	7	8	9
Eigenvalue	3.49	1.65	1.39	0.74	0.5	0.43	0.38	0.23	0.19
Explained Percentage of Variance	38.7	18.3	15.4	8.2	5.6	4.7	4.2	2.6	2.2
Cumulative Explained Percentage of Variance	38.7	57.0	72.5	80.7	86.2	91.0	95.2	97.8	100
**Correlations**									
VABS Communication	0.89	-0.16	0.17	-0.03	0.06	0.0	-0.09	0.16	-0.34
VABS Daily Living Skills	0.85	-0.13	0.19	-0.05	0.0	0.31	-0.05	-0.35	0.02
VABS Socialization	0.87	0.04	0.12	0.05	0.15	0.2	0.27	0.24	0.2
ADI-R Socialization	-0.69	-0.18	0.43	-0.28	-0.11	0.37	-0.24	0.15	0.03
ADI-R Communication	-0.31	-0.34	0.73	-0.32	0.21	-0.21	0.24	-0.08	-0.02
ADI-R Restricted and Repetitive Behavior	-0.17	-0.52	0.45	0.7	-0.07	-0.02	-0.04	0.01	0.02
CBCL Externalizing Problems	-0.14	-0.71	-0.48	-0.03	0.47	0.03	-0.16	0	0.04
CBCL Internalizing Problems	-0.04	-0.78	-0.39	-0.15	-0.38	0.08	0.26	0.01	-0.04
Total IQ	0.78	-0.29	0.12	-0.2	-0.2	-0.32	-0.29	0.04	0.18

Table S4. PCA explained variances and loadings when randomly sampling 30% of SSC patients (n = 823).

**Components**	**1**	**2**	**3**	**4**	**5**	**6**	**7**	**8**	**9**
Eigenvalue	3.53	1.74	1.34	0.76	0.48	0.37	0.37	0.22	0.19
Explained Percentage of Variance	39.2	19.32	14.85	8.40	5.36	4.15	4.10	2.46	2.15
Cumulative Explained Percentage of Variance	39.2	58.53	73.38	81.8	87.14	91.29	95.4	97.8	100
**Correlations**									
VABS Communication	0.89	-0.21	0.15	-0.03	0.05	-0.04	-0.02	0.18	-0.33
VABS Daily Living Skills	0.87	-0.15	0.16	-0.04	-0.06	-0.23	0.09	-0.36	-0.01
VABS Socialization	0.88	0.02	0.10	0.04	0.12	-0.03	0.35	0.19	0.22
ADI-R Socialization	-0.69	-0.27	0.41	-0.27	-0.15	-0.43	0.02	0.13	0.03
ADI-R Communication	-0.31	-0.43	0.69	-0.30	0.24	0.29	0.09	-0.08	-0.01
ADI-R Restricted and Repetitive Behavior	-0.13	-0.57	0.37	0.72	-0.09	0.01	-0.05	0.01	0.03
CBCL Externalizing Problems	-0.16	-0.66	-0.54	0.00	0.47	-0.15	-0.05	-0.01	0.02
CBCL Internalizing Problems	-0.08	-0.73	-0.46	-0.16	-0.39	0.15	0.21	0.00	-0.02
Total IQ	0.78	-0.33	0.07	-0.22	-0.11	0.06	-0.42	0.06	0.18

Table S5. PCA explained variances and loadings when randomly sampling 40% of SSC patients (n = 1098).

**Components**	**1**	**2**	**3**	**4**	**5**	**6**	**7**	**8**	**9**
Eigenvalue	3.45	1.68	1.38	0.72	0.50	0.46	0.38	0.24	0.20
Explained Percentage of Variance	38.35	18.63	15.36	7.97	5.51	5.11	4.22	2.64	2.21
Cumulative Explained Percentage of Variance	38.35	56.98	72.34	80.30	85.82	90.92	95.15	97.79	100
**Correlations**									
VABS Communication	-0.88	0.18	-0.17	0.03	-0.05	-0.02	0.09	-0.20	0.33
VABS Daily Living Skills	-0.84	0.16	-0.21	0.00	-0.03	0.30	0.14	0.35	0.01
VABS Socialization	-0.87	-0.03	-0.12	-0.04	-0.19	0.24	-0.19	-0.22	-0.23
ADI-R Socialization	0.70	0.22	-0.42	0.21	0.11	0.30	0.34	-0.15	-0.05
ADI-R Communication	0.33	0.36	-0.70	0.38	-0.18	-0.12	-0.29	0.07	0.03
ADI-R Restricted and Repetitive Behavior	0.20	0.55	-0.41	-0.69	-0.02	-0.09	0.01	-0.01	-0.03
CBCL Externalizing Problems	0.11	0.68	0.52	0.13	-0.44	-0.08	0.17	0.00	-0.04
CBCL Internalizing Problems	0.03	0.77	0.41	0.07	0.36	0.20	-0.24	-0.02	0.04
Total IQ	-0.76	0.28	-0.14	0.19	0.29	-0.39	0.17	-0.01	-0.17

Table S6. PCA explained variances and loadings when randomly sampling 50% of SSC patients (n = 1372).

**Components**	**1**	**2**	**3**	**4**	**5**	**6**	**7**	**8**	**9**
Eigenvalue	3.46	1.65	1.38	0.75	0.50	0.43	0.38	0.25	0.20
Explained Percentage of Variance	38.48	18.33	15.39	8.37	5.51	4.74	4.25	2.73	2.21
Cumulative Explained Percentage of Variance	38.48	56.80	72.19	80.56	86.08	90.81	95.07	97.79	100
**Correlations**									
VABS Communication	-0.88	0.17	-0.18	0.02	-0.06	0.04	-0.09	0.25	0.30
VABS Daily Living Skills	-0.85	0.14	-0.19	0.04	-0.01	-0.28	-0.15	-0.35	0.06
VABS Socialization	-0.86	-0.03	-0.12	-0.04	-0.20	-0.27	0.17	0.18	-0.25
ADI-R Socialization	0.69	0.15	-0.44	0.27	0.15	-0.28	-0.33	0.15	-0.06
ADI-R Communication	0.32	0.29	-0.74	0.33	-0.25	0.14	0.26	-0.08	0.03
ADI-R Restricted and Repetitive Behavior	0.22	0.46	-0.47	-0.72	0.05	0.02	-0.02	0.00	-0.02
CBCL Externalizing Problems	0.16	0.73	0.46	0.04	-0.42	0.07	-0.22	0.00	-0.06
CBCL Internalizing Problems	0.10	0.81	0.33	0.12	0.30	-0.19	0.28	0.02	0.05
Total IQ	-0.75	0.32	-0.15	0.18	0.30	0.37	-0.13	0.00	-0.18

Table S7. PCA explained variances and loadings when randomly sampling 60% of SSC patients (n = 1646).

**Components**	**1**	**2**	**3**	**4**	**5**	**6**	**7**	**8**	**9**
Eigenvalue	3.47	1.65	1.37	0.75	0.50	0.44	0.39	0.24	0.19
Explained Percentage of Variance	38.56	18.36	15.18	8.36	5.60	4.87	4.30	2.66	2.11
Cumulative Explained Percentage of Variance	38.56	56.92	72.10	80.47	86.06	90.93	95.23	97.89	100
**Correlations**									
VABS Communication	0.89	-0.15	0.16	-0.04	0.07	0.00	0.10	0.19	0.33
VABS Daily Living Skills	0.85	-0.11	0.20	-0.06	-0.02	0.29	0.08	-0.36	0.00
VABS Socialization	0.86	0.03	0.12	0.04	0.14	0.26	-0.25	0.22	-0.21
ADI-R Socialization	-0.68	-0.18	0.43	-0.29	-0.15	0.34	0.28	0.14	-0.04
ADI-R Communication	-0.30	-0.33	0.74	-0.31	0.25	-0.19	-0.25	-0.07	0.02
ADI-R Restricted and Repetitive Behavior	-0.13	-0.53	0.44	0.71	-0.09	-0.01	0.03	0.01	-0.02
CBCL Externalizing Problems	-0.13	-0.72	-0.47	-0.02	0.46	0.04	0.18	0.00	-0.05
CBCL Internalizing Problems	-0.02	-0.78	-0.37	-0.18	-0.37	0.07	-0.27	0.00	0.05
Total IQ	0.78	-0.26	0.12	-0.19	-0.20	-0.37	0.25	0.04	-0.18

Table S8. PCA explained variances and loadings when randomly sampling 70% of SSC patients (n = 1921).

Components	1	2	3	4	5	6	7	8	9
Eigenvalue	3.55	1.63	1.35	0.76	0.51	0.41	0.38	0.22	0.19
Explained Percentage of Variance	39.34	18.1	15.02	8.47	5.63	4.57	4.22	2.49	2.12
Cumulative Explained Percentage of Variance	39.34	57.5	72.50	80.97	86.60	91.16	95.39	97.88	100
**Correlations**									
VABS Communication	0.89	-0.16	0.19	-0.02	0.05	0.00	-0.09	-0.13	0.35
VABS Daily Living Skills	0.86	-0.13	0.18	-0.05	0.04	0.30	-0.01	0.34	-0.03
VABS Socialization	0.87	0.04	0.12	0.05	0.18	0.16	0.28	-0.25	-0.18
ADI-R Socialization	-0.70	-0.16	0.40	-0.31	-0.06	0.40	-0.20	-0.14	-0.02
ADI-R Communication	-0.34	-0.30	0.74	-0.30	0.19	-0.25	0.23	0.08	0.01
ADI-R Restricted and Repetitive Behavior	-0.20	-0.48	0.47	0.71	-0.11	0.02	-0.04	-0.01	-0.03
CBCL Externalizing Problems	-0.14	-0.74	-0.42	0.01	0.48	0.00	-0.16	0.00	-0.04
CBCL Internalizing Problems	-0.04	-0.78	-0.37	-0.16	-0.37	0.06	0.28	-0.01	0.04
Total IQ	0.77	-0.29	0.16	-0.21	-0.23	-0.25	-0.32	-0.05	-0.18

Table S9. PCA explained variances and loadings when randomly sampling 80% of SSC patients (n = 2195).

Components	1	2	3	4	5	6	7	8	9
Eigenvalue	3.45	1.68	1.40	0.73	0.50	0.43	0.38	0.23	0.20
Explained Percentage of Variance	38.28	18.63	15.59	8.10	5.59	4.74	4.25	2.60	2.21
Cumulative Explained Percentage of Variance	38.28	56.91	72.51	80.61	86.20	90.94	95.19	97.79	100
**Correlations**									
VABS Communication	0.89	-0.18	0.15	-0.04	0.06	-0.01	-0.10	-0.13	0.35
VABS Daily Living Skills	0.85	-0.13	0.19	-0.04	0.00	-0.30	-0.03	0.35	-0.04
VABS-Socialization	0.87	0.04	0.12	0.08	0.14	-0.20	0.26	-0.25	-0.19
ADI-R Socialization	-0.67	-0.23	0.42	-0.28	-0.11	-0.38	-0.24	-0.14	-0.03
ADI-R Communication	-0.30	-0.42	0.69	-0.30	0.25	0.21	0.25	0.07	0.01
ADI-R Restricted and Repetitive Behavior	-0.15	-0.57	0.41	0.69	-0.12	0.03	-0.05	-0.01	-0.02
CBCL Externalising problems	-0.11	-0.67	-0.54	0.01	0.47	-0.05	-0.17	0.00	-0.05
CBCL Internalising Problems	-0.02	-0.74	-0.46	-0.18	-0.37	-0.06	0.27	-0.01	0.04
Total IQ	0.77	-0.29	0.11	-0.23	-0.19	0.32	-0.28	-0.05	-0.18

Table S10. Case presentation on baseline.

IDs	VABS Com	VABS Soc	VABS DLS	ADI Soc	ADI Com	ADI RRB	Total IQ	CBCL Int	CBCL Ext	PC1	PC2	PC3	MGFS
ID-09	49	61	54	21	5	6	71	59	56	-0.95	1.03	1.86	8.7
ID-13	52	66	60	18	8	7	73	76	77	-0.75	-0.92	2.58	7.0
ID-16	49	54	56	24	13	10	55	74	71	-1.60	-1.18	1.22	1.8
ID-22	54	61	70	26	12	7	62	70	65	-1.03	-0.49	1.06	3.9
ID-23	63	68	84	24	13	10	51	58	54	-0.53	0.08	-0.27	3.4
ID-25	40	65	54	28	14	3	58	80	77	-1.60	-0.88	2.18	4.3
ID-28	48	53	59	14	10	5	69	73	70	-0.96	-0.22	2.65	8.0
ID-29	50	51	54	28	7	5	54	72	56	-1.55	0.36	1.87	6.5
ID-31	33	49	31	25	14	9	49	72	67	-2.49	-0.59	1.42	1.7
ID-33	47	54	35	28	16	5	49	77	77	-2.18	-0.99	1.82	2.2
ID-39	31	45	32	24	13	10	49	66	51	-2.48	0.29	0.80	2.1
ID-42	50	54	52	20	12	6	50	62	57	-1.39	0.64	1.31	6.3
ID-44	49	74	53	13	13	3	52	53	60	-0.70	1.46	1.64	9.2
ID-47	49	61	49	24	20	8	66	60	64	-1.56	-0.31	-0.02	1.4
ID-48	41	44	35	18	12	7	49	76	69	-2.07	-0.47	2.40	4.7
ID-50	35	50	25	20	8	6	49	62	68	-2.18	0.64	2.57	7.3
ID-51	48	56	45	27	13	3	54	45	56	-1.66	1.75	0.73	6.8
ID-53	45	54	53	25	9	4	68	79	85	-1.51	-1.10	3.05	6.0
ID-54	52	56	50	23	12	3	75	71	66	-1.24	0.008	2.00	6.7
ID-56	49	51	52	23	13	4	67	59	56	-1.41	0.87	1.14	6.4
ID-57	38	37	53	28	14	2	55	70	55	-2.12	0.60	1.52	5.0
ID-59	49	52	47	26	13	8	56	74	62	-1.79	-0.56	1.17	2.5
ID-60	54	66	68	17	7	3	55	81	79	-0.60	-0.62	3.45	9.0
ID-63	42	48	36	23	14	6	49	65	57	-2.09	0.48	1.26	4.2
ID-66	50	56	68	22	14	9	72	70	60	-1.08	-0.60	0.67	2.8
ID-77	47	51	45	24	19	3	70	63	62	-1.71	0.29	0.88	3.8
ID-78	47	57	53	23	20	6	59	65	67	-1.60	-0.38	0.56	2.1
